# Chk2 and P53 Regulate the Transmission of Healed Chromosomes in the *Drosophila* Male Germline

**DOI:** 10.1371/journal.pgen.1004130

**Published:** 2014-02-27

**Authors:** Simon W. A. Titen, Ho-Chen Lin, Jayaram Bhandari, Kent G. Golic

**Affiliations:** Department of Biology, University of Utah, Salt Lake City, Utah, United States of America; The University of North Carolina at Chapel Hill, United States of America

## Abstract

When a dicentric chromosome breaks in mitosis, the broken ends cannot be repaired by normal mechanisms that join two broken ends since each end is in a separate daughter cell. However, in the male germline of *Drosophila melanogaster*, a broken end may be healed by *de novo* telomere addition. We find that Chk2 (encoded by *lok*) and P53, major mediators of the DNA damage response, have strong and opposite influences on the transmission of broken-and-healed chromosomes: *lok* mutants exhibit a large increase in the recovery of healed chromosomes relative to wildtype control males, but *p53* mutants show a strong reduction. This contrasts with the soma, where mutations in *lok* and *p53* have the nearly identical effect of allowing survival and proliferation of cells with irreparable DNA damage. Examination of testes revealed a transient depletion of germline cells after dicentric chromosome induction in the wildtype controls, and further showed that P53 is required for the germline to recover. Although *lok* mutant males transmit healed chromosomes at a high rate, broken chromosome ends can also persist through spermatogonial divisions without healing in *lok* mutants, giving rise to frequent dicentric bridges in Meiosis II. Cytological and genetic analyses show that spermatid nuclei derived from such meiotic divisions are eliminated during spermiogenesis, resulting in strong meiotic drive. We conclude that the primary responsibility for maintaining genome integrity in the male germline lies with Chk2, and that P53 is required to reconstitute the germline when cells are eliminated owing to unrepaired DNA damage.

## Author Summary

Using the fruitfly as an experimental model system, we produced chromosomes that were broken at one end, and thus lacked the normal telomeric cap. The occurrence of such chromosomes is thought to promote carcinogenesis. A cell's response to such chromosomes is therefore of great interest. In somatic cells the tumor suppressors Chk2 and P53 can induce suicide of cells with such a chromosome and eliminate the danger. In the male germline, though, such chromosomes can be healed by the addition of a new telomere cap, and may then be transmitted to the next generation. We find that Chk2 and P53 regulate healing in the germline, but in seemingly opposite directions. Chk2 functions independently of P53 to eliminate cells with a damaged chromosome, while P53 is required to repopulate the germline after this Chk2-mediated elimination. Cells that carry a broken chromosome continue to divide in Chk2 mutants. We observed that the broken ends of sister chromatids may fuse in meiosis and that the fused chromatids produce a bridge spanning two cells at the second meiotic division. This structure elicits a previously undiscovered mechanism to eliminate sperm derived from such cells, providing an added safeguard to maintain genome integrity through the germline.

## Introduction

Barbara McClintock discovered that dicentric chromosomes produced in germ cells of corn plants could break, and that the broken chromosomes could be transmitted and have a new telomere added to the broken end. She called this process healing [1], [2]. Extensive early investigations in Drosophila led to the conclusion that chromosomes could not be healed in this way, and it seemed that this might indicate a fundamental difference between plants and animals [3], [4]. However, in the last several decades a number of examples of broken and healed chromosomes have been identified in animals, making it clear that healing can occur in a variety of species, including humans [5]–[10].

**Figure 1:**
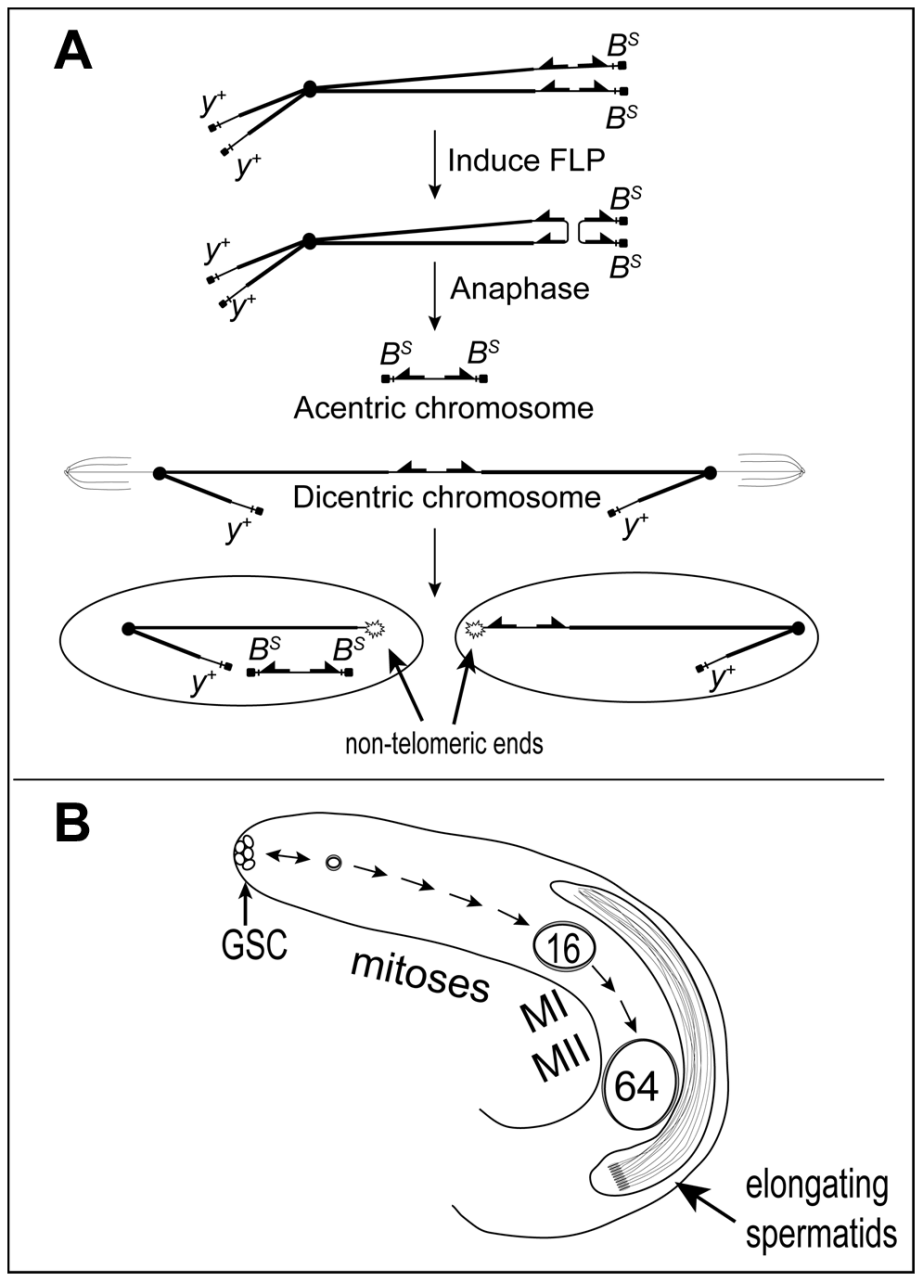
Dicentric chromosome formation and spermatogenesis. A. Mechanism to generate a dicentric *Y* chromosome. FLP catalyzes recombination between inverted *FRT*s on sister chromatids of a *Y* chromosome marked with *Bar ^Stone^* (*B^S^*) and *yellow^+^* (*y^+^*) to produce a dicentric chromosome marked with *y^+^* and an acentric chromosome carrying both copies of *B^S^*. During mitosis, breakage of the dicentric at a non-central site produces a short centric fragment *Y* lacking *B^S^* and *FRT*s and a long centric fragment *Y* lacking *B^S^* but carrying inverted *FRT*s. The acentric chromosome is not expected to segregate reliably. B. Overview of early spermatogenesis in the *Drosophila melanogaster* testis. Germline stem cells (GSC) at the apical tip divide asymmetrically to produce another stem cell and a primary spermatogonial cell, which becomes surrounded by two somatic cyst cells which do not divide further. A spermatogonial cell normally undergoes four rounds of mitosis followed by the two meiotic divisions to produce a cyst of 64 haploid spermatids. After meiosis the spermatids differentiate and elongate, followed by individualization and release of mature sperm into the seminal vesicle (not shown).

Dicentric chromosomes can be efficiently produced in *Drosophila melanogaster* by FLP-mediated recombination between *FRT*s in opposite orientation on sister chromatids (Figure 1A) [11]. Such chromosomes typically break in the subsequent mitotic division, delivering a chromosome with a single broken end to each daughter cell [12]. We sometimes refer to such damage as telomere loss, since it is unrepairable by normal mechanisms that join two broken ends, but may be healed by *de novo* addition of a new telomere cap [6], [13]. In the work reported here we assayed the frequency of chromosome healing using a *Y* chromosome, *DcY(H1)* or simply *H1*, marked with the dominant genes *B^S^* on the long arm and *y^+^* on the short arm. A *P* element insertion, *P{iw}*, carrying inverted copies of the FLP Recombination Target (*FRT*) lies proximal to *B^S^* on the long arm, allowing for FLP-mediated generation of dicentric chromosomes. In a testcross, progeny that receive a broken-and-healed *H1* chromosome may be recognized as those that have lost *B^S^* but retain *y^+^*. The use of a *Y* chromosome avoids complications owing to aneuploidy that might result if dicentrics were produced on the *X* or an autosome [12].

Spermatogenesis occurs continuously throughout the life of *Drosophila melanogaster* males (Figure 1B; reviewed by [14], [15]). Primary spermatogonial cells (*aka* gonialblasts) are produced by the asymmetric division of stem cells at the apical tip of the testis. Each becomes enclosed by two somatic cyst cells, and subsequent development occurs synchronously for cells within a single cyst. The primary spermatogonial cell undergoes four mitotic divisions to produce a cyst carrying 16 primary spermatocytes, followed by two meiotic divisions. All divisions within a cyst occur without complete cytokinesis, to generate 64 interconnected haploid spermatids. The 64 sperm heads remain tightly clustered during post-meiotic spermatid differentiation until they are individualized and released into the seminal vesicle. Induction of dicentric chromosome formation and breakage in the testis allows us to combine cytological observations of the tissue and individual cells, with crosses that can reveal the ultimate fate of such cells.

In somatic cells of Drosophila, dicentric chromosome breakage activates key proteins of the DNA damage response (DDR) and, via the Chk2 checkpoint kinase (encoded by *lok*) and the P53 tumor suppressor homolog (*p53*), leads most cells into apoptosis [12], [16]. We examined the roles of these genes on the process of chromosome healing in the male germline.

## Results

### Chk2 and P53 have strong and opposite effects on transmission of healed chromosomes

Males carrying the *H1* chromosome and a heat shock-inducible *FLP* transgene (*70FLP*) were heat-shocked during the first 24 hours of development and adults that eclosed were test-crossed to score progeny carrying broken-and-healed *Y* chromosomes (referred to as Fragment *Y* chromosomes, or *FrY*; Table 1). In control matings, heat-shocked *70FLP/H1* males transmitted an *FrY* chromosome to 11% of their sons (indicated as Fragment Ratio, or FR), but *lok* males transmit *FrY* chromosomes at the much higher rate of 90% (P<0.0001). Thus, Chk2 must normally limit the transmission of broken-and-healed chromosomes. We also found that *lok* is haplo-insufficient, with *lok/+* heterozygotes showing intermediate values of 67% or 28% fragment transmission (P<0.0001 for both vs. “wildtype” control). The difference in these two results owes to whether the mothers of tested males were homozygous or heterozygous for *lok* (respectively), reflecting a maternal contribution [17]. Similarly, *lok* males carrying a *lok^+^* transgene transmitted 60% *FrY* chromosomes, significantly fewer than *lok* homozygotes without the complementing transgene (P = 0.0004), but more than wildtype males (P<0.0001).

**Table 1:** *FrY* recovery from wildtype and mutant males (38° 1 hr. heat shock at 0–24 hours of development).

	male progeny						
tested males	*Y*	*FrY*	female progeny	FR	SR	N	% fertile
a +	4496	566	5845	0.11	0.87	205	64
b *lok*	151	1372	3542	0.90	0.43	112	65
c *lok* ^+/−^	391	786	1240	0.67	0.95	40	65
d *lok* ^+/−^	969	390	1661	0.28	0.82	52	90
e *lok; {lok^+^}*	782	1185	2367	0.60	0.83	225	31
f *p53*	1119	13	1332	0.011	0.85	136	23
g *p53*	2190	152	2739	0.065	0.86	578	16
h *p53; {p53^+^}*	2181	491	3148	0.18	0.85	414	23
i *lok; p53*	113	914	2221	0.89	0.46	136	52

Males were testcrossed individually to *y w* females. FR = fragment ratio calculated as *FrY/(FrY + Y)* sons; SR = sex ratio calculated as (total male progeny)/(total female progeny); N, total males testcrossed; % fertile is fraction of testcrossed males that produced any progeny. Genotypes of males tested:

a *y w 70FLP*3F/*DcY, H1.*

b *y w 70FLP*3F/*DcY, H1; lok^P6^* (from *lok^P6^* homozygous mothers).

c *y w 70FLP*3F/*DcY, H1; lok^P6/+^* (from *lok^P6^* homozygous mothers).

d *y w 70FLP*3F/*DcY, H1; lok^P6/+^* (from *lok^P6/+^* heterozygous mothers).

e *y w 70FLP*3F/*DcY, H1; lok^P6^; P{lok^+^}^AM12^/+* (from *lok^P6/+^* heterozygous mothers).

f *y w 70FLP*3F/*DcY, H1; p53^5A-1-4^* (from *p53^5A-1-4^* homozygous mothers).

g *y w 70FLP*3F/*DcY, H1; p53^5A-1-4^* (from *p53^5A-1-4/+^* heterozygous mothers).

h *y w 70FLP*3F/*DcY, H1; P{p53^+^, ry^+^}3A/+; p53^5A-1-4^* (from *p53^5A-1-4^* homozygous mothers).

i *y w 70FLP*3F/*DcY, H1; lok^P6^; p53^5A-1-4^* (from *lok^P6/+^; p53^5A-1-4/+^* heterozygous mothers).

We next tested the effect of *p53* on germline fragment transmission. In the soma of *p53* flies, as with *lok* flies, cells with a broken chromosome exhibit increased survival [12], [16]. This is expected since P53 is activated by Chk2, and P53 is largely responsible for the rapid apoptotic response to DNA damage [18]–[24]. Surprisingly, we found that the germline effect of *p53* was opposite that of *lok*: *FrY* transmission from *p53-*null males (*p53^−/−^* sons of *p53^−/−^* mothers) dropped to 1.1% (P = 0.033). Homozygous sons of *p53/+* heterozygous mothers had a slightly higher rate of fragment transmission of 6.4%, indicating a maternal contribution, though this was still lower than the 11% seen in *p53^+^* males (P = 0.018). Finally, the addition of a *p53^+^* transgene to *p53* males reversed the reduction in fragment transmission (P = 0.02), with such males showing an even higher rate of transmission (18%) than the wildtype control, though not significantly so (P = 0.45).

We also examined the effect of *lok* and *p53* mutations on fragment transmission in males carrying an extra copy of *YL* (attached to the *X* chromosome) using an alternate heat shock protocol. Fragment transmission from the control males was 53.0%, which increased to 96.0% from *lok* males (P<0.0001) and decreased to 9.0% from *p53* males (P<0.0001), confirming the effects of these mutations (Table 2).

**Table 2:** *FrY* recovery from wildtype and mutant males (38° 1 hr. heat shock at 0–72 hours of development).

	male progeny						
tested males	*Y*	*FrY*	female progeny	FR	SR	N	% fertile
a +	1894	2124	4245	0.53	0.95	426	25
b *lok*	71	1627	3520	0.96	0.48	142	49
c *p53*	3092	312	3529	0.09	0.96	1385	9.5

Males were testcrossed individually to *y w* females. Genotypes of males tested:

a *y w 70FLP*3F•*YL/DcY, H1*.

b *y w 70FLP*3F•*YL/DcY, H1; lok^P6^* (from *lok^P6/+^* heterozygous mothers).

c *y w 70FLP*3F•*YL/DcY, H1; p53^5A-1-4^* (from *p53^5A-1-4^* homozygous mothers).

### P53 is required for recovery of the male germline following induction of dicentric chromosomes

**Figure 2:**
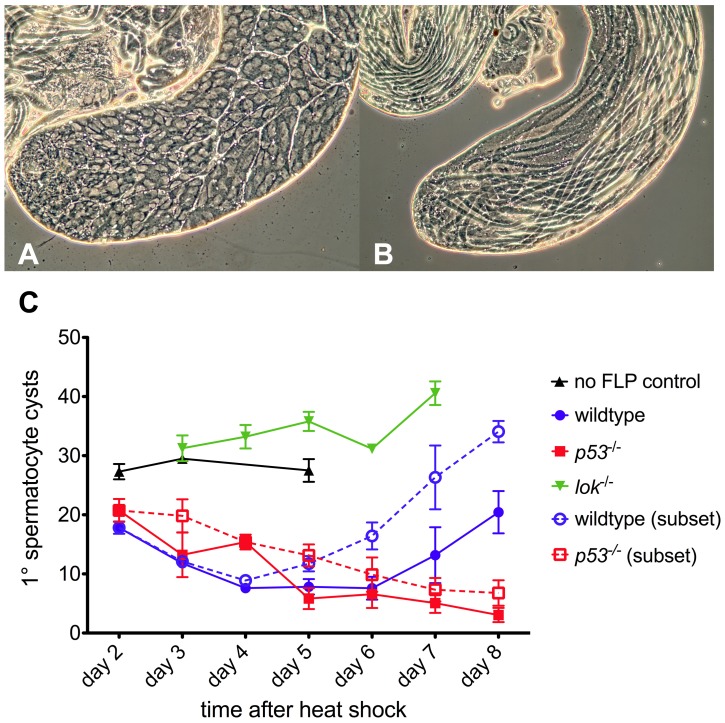
Primary spermatocyte cysts following dicentric chromosome induction. Phase contrast views of a normal testis and *yw/DcY(H1); hsFLP*2B/+ testis five days after heat shock. The apical portion of a normal testis (A) is filled with cysts, with primary spermatocyte cysts occupying most of the volume. Stem cells are located at the left tip. After dicentric induction (B) very few primary spermatocyte cysts are found (none in this particular testis). Instead, elongating spermatid cysts, derived from cells which were beyond the heat shock responsive stage [65], [66], occupy the entire length of the testis. (C) The primary spermatocyte cyst population after heat shock induction of dicentric chromosomes. Flies that do not make dicentrics (no FLP control, ▴) show no reduction of primary spermatocyte cysts after heat shock. After dicentric induction there is a reduction in primary spermatocyte cysts, followed by recovery in wildtype males (•), but not in *p53* mutants (▪). The *lok* mutant males (▾) showed no reduction in primary spermatocyte cysts after dicentric induction. Dotted lines with open symbols represent data only for testes that had at least one primary spermatocyte cyst. Error bars indicate ±1 SEM.

The *p53* mutant males were substantially more sterile than control males (Tables 1, 2). To investigate the nature of this sterility we undertook a cytological investigation of the male germline after dicentric chromosome induction. We dissected testes of newly eclosed adult males at sequential times after heat-shock induction of *FLP* and scored the population of primary spermatocyte cysts (Figure 2). Control males (*i.e.*, not making dicentrics because they did not carry *hsFLP*) that were heat-shocked exhibited no significant change in spermatocyte population from days 2–5, showing that heat shock alone has little effect. In wildtype and *p53* males, after induction of dicentric chromosome formation, the number of primary spermatocyte cysts decreased from ∼20 per testis at two days after heat shock, to an average of ∼7–8 per testis at 4–6 days after heat shock. The germlines of wildtype males showed a strong recovery over the next two days, but in *p53* mutant males the number of primary spermatocyte cysts continually decreased, showing no recovery through the length of the experiment. Both *p53* and *lok* males had numerous testes with no primary spermatocyte cysts, averaging 44% for days 5–8 for both genotypes, indicating that the germline was completely ablated in nearly half of the testes of both genotypes. Even when the testes that completely lack primary spermatocytes were removed from consideration, *p53* males still showed no recovery, while wildtype males showed robust recovery (Figure 2C, dotted lines). In contrast, *lok* mutant males showed a more or less continual increase in the primary spermatocyte population throughout the course of our examination. We conclude that Chk2 normally restricts the survival or growth of germline cells with a broken chromosome, reducing the germline population, while P53 is required for the germline to recover from this reduction.

### Post-meiotic spermatid elimination causes sex ratio distortion in *lok* males

Although control and *p53* males produced slightly fewer sons than daughters after induction of *Y* chromosome dicentrics, *lok* males had many fewer, producing only about half as many sons as daughters. This sex ratio (SR) distortion must be principally a consequence of *Y* chromosome dicentric formation, since non-heat-shocked *lok* males did not show this dramatic reduction in sons compared to *lok^+^* males (Table 3). The reduced recovery of sons implies that *Y-*bearing gametes are eliminated after meiosis, because if cells with a *Y* were eliminated prior to meiosis then *X*- and *Y*-bearing sperm should be reduced equally.

**Table 3:** *FrY* recovery: No heat shock controls.

	male progeny						
tested males	*Y*	*FrY*	female progeny	FR	SR	N	% fertile
a +	3215	0	3482	0.00	0.92	66	100
b *lok*	2660	5	3186	0.002	0.84	73	92
c *p53*	206	0	307	0.00	0.67	14	43

Males were testcrossed individually to *y w* females. Genotypes of males tested were:

a *y w 70FLP*3F/*DcY, H1*.

b *y w 70FLP*3F/*DcY, H1; lok^P6^* (from *lok^P6^* homozygous mothers).

c *y w 70FLP*3F/*DcY, H1; p53^5A-1-4^* (from *p53^5A-1-4^* homozygous mothers).

One explanation for this sex ratio distortion could be that *lok* males transmit sperm carrying an uncapped *Y* chromosome, and this produces zygotic lethality. To test this we scored egg-to-adult viability of zygotes produced by *y w 70FLP/H1; lok* males that had been heat-shocked, or not, to induce FLP synthesis and dicentric formation (Table 4). We observed very little zygotic lethality in these crosses. Even though these heat-shocked males exhibit strong meiotic drive, with sex ratios of 0.22 and 0.27, lethality among their offspring increased only 4–5% relative to non-heat-shocked males. If *lok* males transmit any broken chromosomes that act as dominant lethals it must be at a low level, and is insufficient to account for the observed sex-ratio distortion.

**Table 4:** Viability of eggs fertilized by *y w 70FLP3F/DcY, H1; lok/lok* males.

Heat shock	Eggs	Adults	Survival (%)	FR	SR
a −	492	447	91	0	1.02
a +	646	554	86	0.6	0.27
b −	716	612	85	0.004	0.89
b +	458	371	81	0.79	0.22

*w^1118^/H1; lok^P6^* males were crossed to either.

a *y w 70FLP; lok^P6^/Cy lok^+^*, or

b *y w 70FLP; lok^P6^/lok^P6^* females and their progeny were heat-shocked (or not) at 38° for one hour during the first 24 hrs. of development. The *y w 70FLP/H1; lok/lok* males that eclosed were then crossed to *y w* females and egg to adult survival of their progeny was scored.

FR, fragment ratio; SR, sex ratio.

We examined testes of these males to see whether we could detect any abnormalities that might account for sex-ratio distortion. *70FLP/H1* males were heat-shocked during the first 24 hours of development and then dissected within 24 hours of eclosing as adults. One immediately obvious difference between *lok^+^* and *lok* males was that many of the *lok^+^* males had vestigial or absent testes (24% of 187 males examined missing one or both testes), while all *lok* males had the expected two testes (47 males examined; P<0.0001).

**Figure 3:**
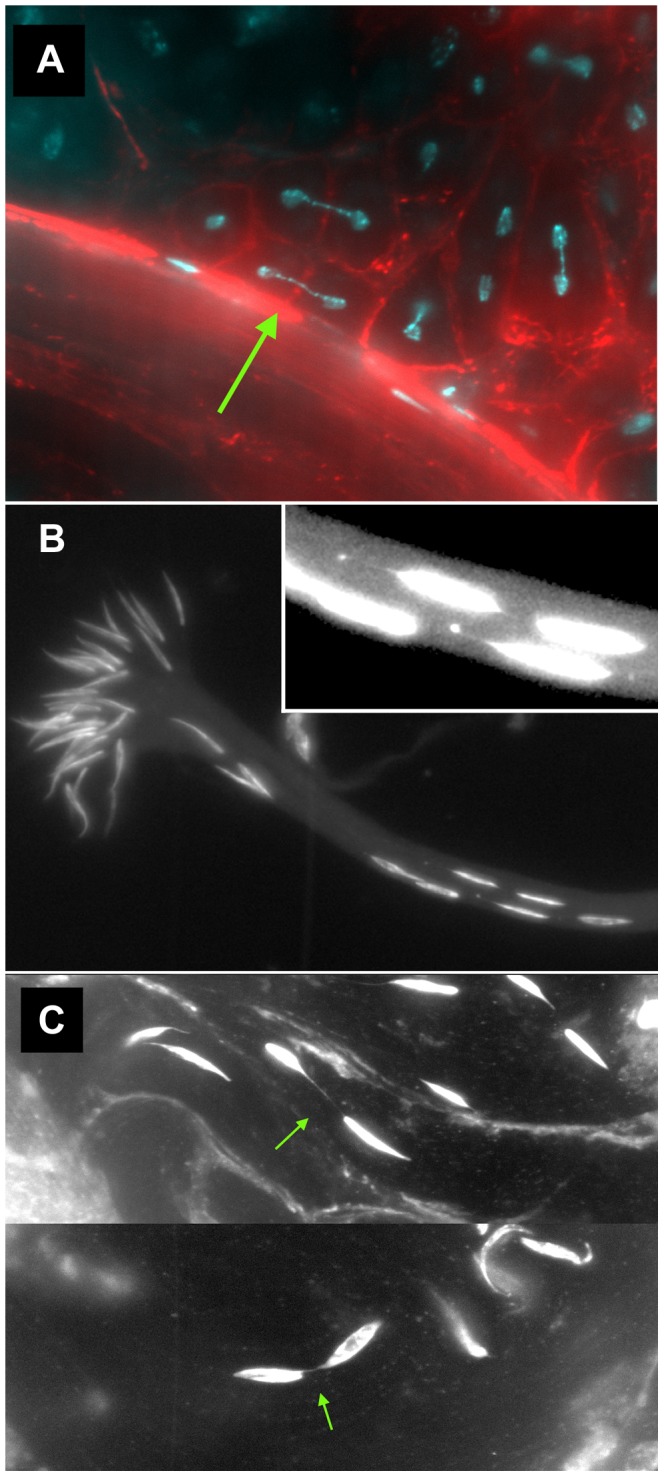
Meiotic and spermatid phenotypes of *lok* males after dicentric chromosome induction. *y w 70FLP*3F/*DcY(H1)*; *lok^P6^* males were heat-shocked at 38° for 1 hr during the first 24 hours of development, then dissected within 24 hrs of eclosion and stained with DAPI (blue) and phalloidin coupled to rhodamine or FITC (red). (A) *Y* chromosome dicentric bridges were frequently observed in MII, even in cells with near complete cytokinesis (arrow). (B) Sperm heads were often displaced from the bouquet of differentiating heads. The displaced heads were frequently mis-shapen, with some showing threads of trailing chromatin (inset - brightness increased to aid visualization). (C) Displaced sperm heads were sometimes connected by thin chromatin bridges.

We also found numerous examples of two specific anomalies in *lok* males after dicentric induction. First, we observed frequent dicentric bridges in Meiosis II involving the *Y* chromosome (as judged by their strongly banded appearance; Figure 3A). Since FLP synthesis was induced ∼10 days prior to dissection, at a time when only cells in the very earliest stages of spermatogenesis were present, we interpret these bridges as evidence that chromosomes with broken ends persist through several mitotic divisions in *lok* mutants, with broken ends of sister chromatids fusing prior to MII. Though we did not attempt to identify bridges in mitoses of spermatogonial divisions, it seems likely that such chromosomes were undergoing bridge-breakage-fusion cycles in the preceding mitotic divisions as well.

**Figure 4:**
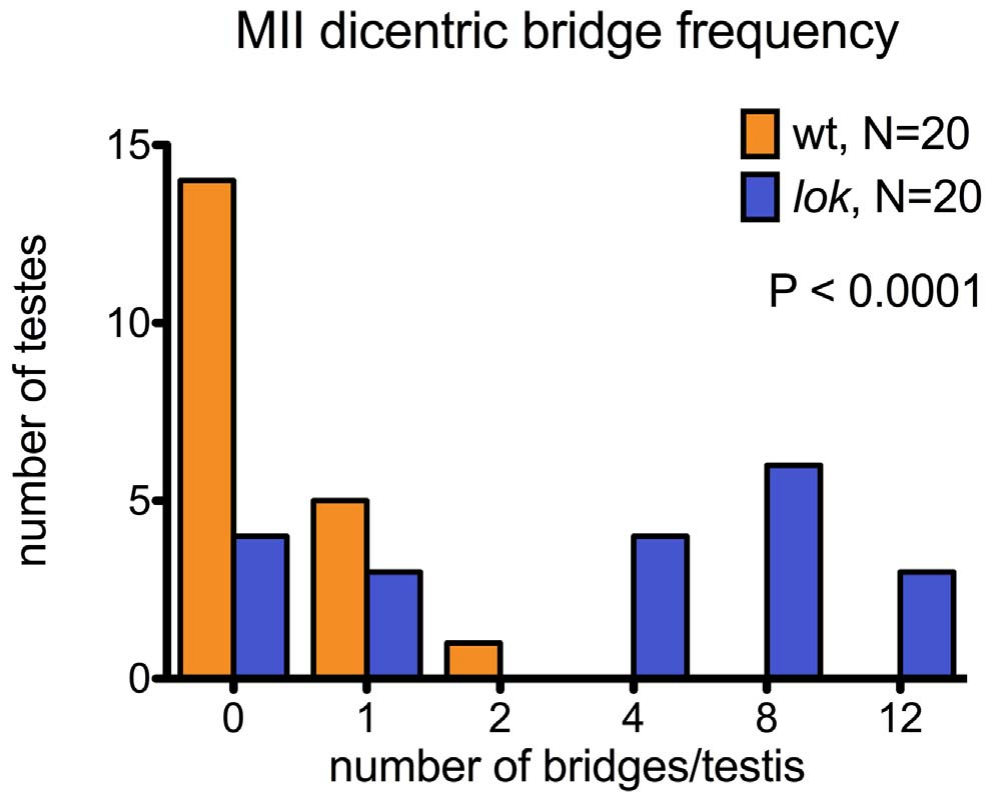
Dicentric bridge frequency in Meiosis II. MII dicentric bridges were scored in testes dissected from wildtype or *lok* males, using the same protocol as for Figure 3.

In *lok* males, there were cases where a majority of the presumed 16 *Y*-bearing MII divisions within a cyst had dicentric bridges (Figure 4 — cysts with 12 MII bridges). We also observed occasional chromatin bridges in Meiosis II divisions of *lok^+^* testes after dicentric induction, indicating that even in wildtype males some cells continued to divide with an unrepaired broken chromosome end. However, such bridges were much less frequent than in *lok* males (Figure 4; P<0.0001).

These testes were also stained with phalloidin to visualize F-actin of the cytoskeleton underlying cell membranes. We saw many examples of unbroken dicentric bridges, even in cells where the MII division appears to be complete, as indicated by near-complete cytokinesis (Figure 3A). In these fixed preparations it is not possible to conclude with certainty that MII bridges do not break, and there were some instances of what appeared to be chromatin bridges that had stretched and broken, though they were relatively infrequent. However, our observations (below) of later stages of spermiogenesis lead us to conclude that in many cases such chromatin bridges persist long after meiosis.

A second anomaly observed in the testes of *lok* males after dicentric induction was abnormally located sperm heads in differentiating sperm bundles. The 64 post-meiotic sperm heads of a single cyst are normally clustered into a tight bouquet, with the sperm tails extending towards the apical tip of the testis. But in *lok* males we observed large numbers of sperm heads that were displaced caudally from their normal location, often showing abnormal morphologies (Figure 3B, C). The displaced sperm heads were found at varied locations within any single cyst, ranging from a short distance behind the bouquet of sperm heads all the way to the caudal tip. In *lok* ^+^ males that had experienced dicentric induction we observed an average of 61.2 sperm heads in their normal location, and only 2.8 displaced caudally. However, in *lok* males we found only 43.2 sperm heads in the bouquet and 16.9 that were displaced (Table 5; P<0.0001 for *lok* vs. *lok^+^*). We note that if the ∼17 displaced sperm heads all carried a *Y* chromosome their absence from the population of functional sperm would almost precisely account for the sex ratio distortion seen in such males (32–17/32 = 0.46, *cf*. SR of 0.43 in Table 1).

**Table 5:** Sperm head displacement following dicentric chromosome induction.

genotype	treatment	N	in bouquet	displaced	total
*y w 70FLP/H1*	+ HS	16	61.2±1.6	2.8±1.1	64.0
*y w 70FLP/H1; lok*	+ HS	17	43.2±2.4	16.9±2.7	60.1
*y w/H1*	− HS	20	61.7±0.7	1.8±0.4	63.5
*y w/H1; lok*	− HS	21	53.9±2.4	7.0±2.0	60.9

HS, heat shock; N, number of elongated post-meiotic cysts scored.

The average total number of sperm heads found in all *lok* bundles (with and without *70FLP)* was only 60.5, rather than the expected 64. Although it is difficult to trace a single bundle of differentiating spermatids and score sperm heads along its entire length, we suspect that this reflects a real reduction in the actual number of spermatids in *lok* cysts, since *lok^+^* males had an average of 63.7 sperm heads per cyst (P = 0.0073 for *lok* vs. *lok^+^*). Furthermore, even in the absence of dicentric induction, the *lok* males had many more displaced sperm heads than the comparable *lok^+^* males (7.0 vs. 1.8; P = 0.007). This may reflect the important role that Chk2 plays in quality control during spermatogenesis.

We saw many examples where thin strands of DAPI-staining material connected two displaced sperm heads (Figure 3C), most likely resulting from MII bridges that persisted into spermiogenesis without breaking. We also saw sperm heads that trailed strings and dots of chromatin (Figure 3B, inset), possibly indicative of chromatin bridges that broke during spermatid differentiation.

When the genetic and cytological observations are considered together they lead to the conclusion that MII anaphase chromosome bridges disrupt the subsequent development of spermatids derived from these nuclei, resulting in their elimination from the population of functional gametes. Since such bridges occur frequently on the *Y* chromosome in the *lok* males of these experiments, the sex ratio among their progeny is strongly distorted in favor of females.

### When does healing occur?

In corn, the broken fragments of a dicentric chromosome may be transmitted through the gametophyte, but are healed in the sporophyte, after fertilization [1]. Similarly, it was proposed that in *Drosophila mu-2* females, broken chromosomes may be passed through the oocyte and healed in the zygote after fertilization [25]. However, our observation that a male's genotype influences his transmission of healed chromosomes is most consistent with the interpretation that healing occurs in that male, rather than in his offspring after fertilization. This is further supported by a number of experimental observations.

**Figure 5:**
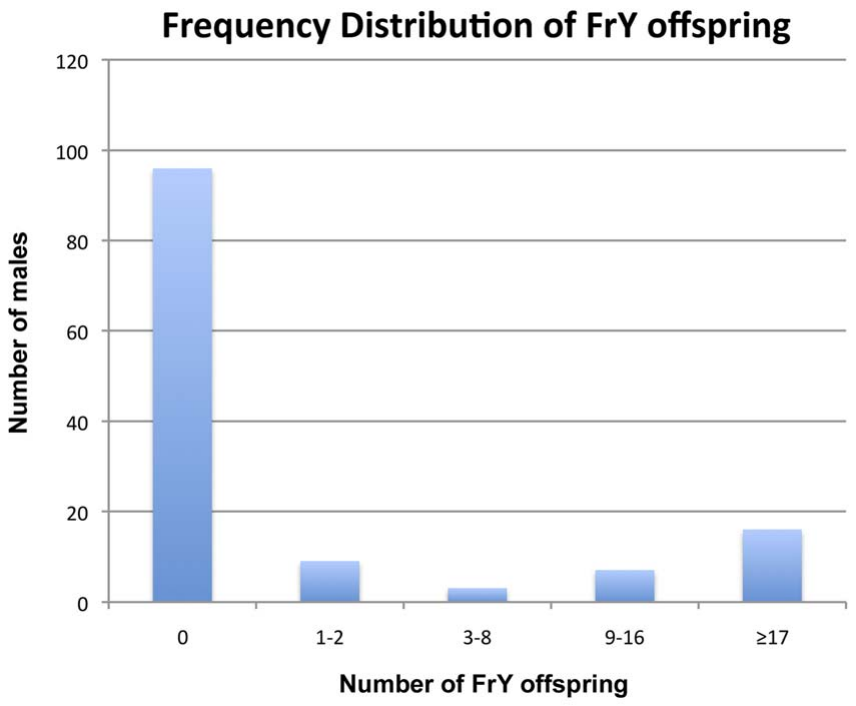
Frequency distribution of *FrY* offspring produced by individual heat-shocked *y w 70FLP/DcY, H1* males.

First, when multiple *FrY* progeny are produced by a wildtype male they appear to represent the clonal expansion of a single infrequent healing event. Although dicentric chromosome formation is very efficient after heat-shock induction of *70FLP* (as judged by >90% rate of *FrY* transmission from *lok* males, and many other evidences [11], [12], transmission of *FrY* chromosomes from wildtype males was relatively infrequent, indicating that in most cells, the broken chromosomes did not heal and the cells were eliminated. The distribution of *FrY* transmission rates indicates two qualitatively distinct classes of male: many males that transmit no *FrY* chromosomes (97), and a smaller number that typically produce multiple *FrY* progeny (35 males with an average of 14.7 *FrY* progeny; Figure 5). To test whether these “jackpots” of *FrY* offspring are copies of a single healed chromosome we asked whether the *FrY* chromosomes transmitted by a single male were the same type, or a mixture of different types. We expect dicentric breakage, unless it occurs very near the point of sister chromatid fusion, to produce one long and one short fragment. If healing occurred after fertilization then we would have expected to recover a mixture of long and short *FrY* chromosomes from any particular male. Instead, we found that nine of 10 wildtype males transmitted only a single type of *FrY* (*P*<0.001; Table 6), supporting the proposition that all *FrY* chromosomes from an individual wildtype male usually derive from a single progenitor cell in which a broken chromosome was healed, and then underwent mitotic expansion.

**Table 6:** Long and short *FrYs* produced by individual males.

fragments from wildtype males			fragments from *lok* males		
father #	long	short	father #	long	short
1	2	4	1	7	0
2	2	0	2	1	5
3	0	6	3	7	0
4	0	9	4	3	1
5	3	0	5	4	6
6	0	9	6	1	6
7	0	2	7	6	4
8	0	9	8	4	1
9	0	8	9	3	6
10	6	0	10	2	2
11	0	4	11	0	5
12	9	0	12	5	5
			13	3	1
totals	22	51	14	0	4
			15	2	1
			16	1	3
			17	4	1
			18	2	0
			totals	55	51

It might be argued that, because long *FrY* chromosomes still carry inverted *FRT*s, repeated rounds of recombination and dicentric breakage could generate a predominance of short fragments that lack *FRT*s. Alternatively, since short fragments might lack *Y*-encoded fertility factors, one could also argue that there is a selection in favor of long fragments. In fact, we found that wildtype males transmitted both types, with six males transmitting only short *FrY* chromosomes, three males transmitting only long *FrY* chromosomes, and one male transmitting both types. We also note that, in contrast to wildtype males, most *lok* males (13/18; Table 6) transmitted both long and short fragments. Although the number of *lok* males transmitting a single type is still higher than expected by chance (*P* = 0.009), there are many more *lok* males that transmit both types when compared to wildtype (*P = *0.001). This likely indicates that multiple independent healing events occurred in most *lok* males.

A second point suggesting that healing occurs in the male germline is that, although *lok* and *p53* both exert strong paternal influence on the recovery of *FrY* chromosomes, these mutations have very little effect in the females to which these males are mated, indicating that they are not acting maternally to influence healing of broken chromosomes in zygotes (Table 7; wildtype vs. *p53* P = 0.31; wildtype vs. *lok* P = 0.99).

**Table 7:** Effect of maternal genotype on *FrY* recovery (38 1 hr. heat shock at 0–24 hours of development).

	male progeny						
maternal genotype	*Y*	*FrY*	female progeny	FR	SR	N	% fertile
+	825	292	1237	0.26	0.90	39	0.85
*lok*	580	225	614	0.28	1.31	51	0.75
*p53*	1322	570	2131	0.30	0.89	54	0.69

*y w 70FLP*3F*/DcY, H1* males were testcrossed individually to *y w* females, or *y w; lok^P6^* females or *y w; p53^5A-1-4^* females.

**Figure 6:**
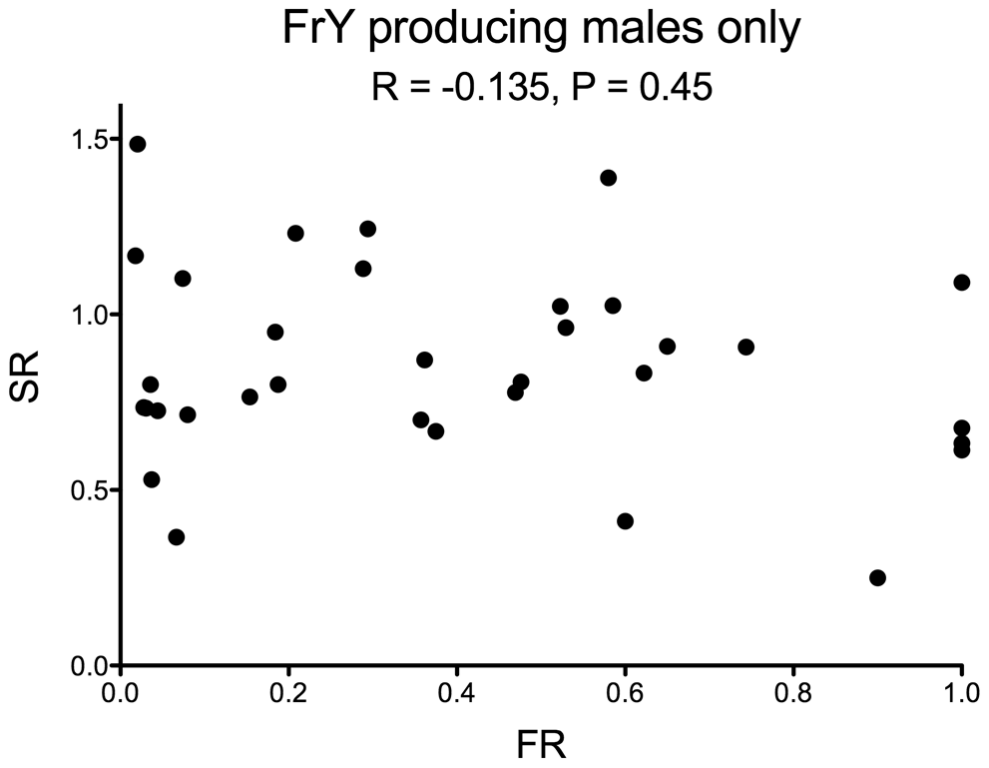
Fragment ratio (FR) vs. Sex Ratio (SR) of individual males that produced any *FrY* offspring (heat-shocked *y w 70FLP/DcY, H1* males). There is no correlation between the two metrics (R = −0.135, P = 0.45). One male that produced 53 *FrY-*bearing sons and three regular daughters was excluded from this graph.

Finally, the rarity of MII chromosome bridges in wildtype males suggests that cells with broken chromosomes do not often reach meiosis in such males. The strong sex-ratio distortion that was seen in *lok* males is not seen with wildtype males, supporting this conclusion. Nor is it always the case that wildtype males eliminate cells with broken chromosomes too efficiently to detect an altered sex ratio that might be produced by unhealed chromosomes reaching meiosis. If we examine only those wild type males from Table 1 that produced any *FrY* offspring (giving an average FR of 0.40; Figure 6), the SR among their progeny is little different from males that did not produce *FrY* offspring (0.84 vs. 0.89 respectively; P = 0.34). And, even in cases where *FrY* chromosomes accounted for 100% of the *Y-*bearing offspring from wildtype males [13], the sex ratio was only slightly lower than in the wildtype males of the experiments reported here (0.81 vs. 0.87, respectively). Taken together, the simplest interpretation of our results is that chromosome healing, when it does occur, occurs prior to meiosis in the male germline.

## Discussion

Our results show that Chk2 and P53 profoundly influence the recovery of broken-and-healed chromosomes through the male germline, but that their effects are quite different. Males that lacked Chk2 showed ∼10-fold increase in *FrY* transmission, while males that lacked P53 showed ∼10-fold decrease. Although P53 has often been called the “guardian of the genome” [26], these results indicate that it is Chk2, acting independently of P53, that is predominantly responsible for preventing transmission of broken-and-healed chromosomes through the male germline in Drosophila.

Chk2 might directly influence healing by repressing a mechanism that builds new telomere caps on broken ends, but we believe that an indirect effect is more likely. In early *Drosophila* embryos and imaginal tissues, in yeast cells, and in mammalian cells, Chk2 blocks cell cycle progression in response to DNA damage [18], [27]–[33]. Our results show that Chk2 functions similarly in the *Drosophila* male germline since, in the absence of Chk2, cells carrying an unrepaired DSB continue to divide. Wildtype males exhibit a transient depletion of germline cysts following dicentric induction, but *lok* males do not. In addition, frequent MII anaphase chromatin bridges in *lok* mutant males show that many cells reach meiosis with broken chromosomes that have not healed, even 9–10 days after *FLP* expression was induced. We suggest that, in the germlines of *lok* males, broken chromosomes have a higher rate of healing simply because they have a longer period (or number of cell cycles) during which healing can occur. Others have similarly proposed that persistence of a non-telomeric end over time may be a critical factor in chromosome healing [34].

Our results point to the existence of a Chk2-independent mechanism that can eliminate spermatids produced from cells with MII chromosome bridges. When such bridges occur specifically on *Y* chromosomes, strong meiotic drive is produced which is seen as a deficiency of sons. The removal of spermatids with this type of chromosome aberration provides another level of genome quality control prior to the production of a functional gamete. This mechanism is also independent of P53, since *lok; p53* double mutant males exhibit the same strong drive (Table 1).

Contrary to its role in somatic cells, P53 is not required to eliminate germline cells following dicentric chromosome induction. In fact, *p53^+^* males transmit *FrY* chromosomes at a higher rate than *p53* mutants. P53 is best known as a transcriptional regulator [35], though it has other functions [36]–[39]. In response to a broken chromosome end in the male germline, P53 might normally promote the expression of genes that mediate healing, repress genes that antagonize healing, or perhaps directly interact with a DSB to mediate healing [40], [41]. However, if P53 were required for healing then *lok; p53* double mutants should also exhibit a low rate of healing, but this was not found. The *lok; p53* double mutant males transmit healed chromosomes at almost the same rate as *lok* by itself (Table 1), indicating that P53 is not needed to heal a broken end.

It is certainly puzzling that *lok* and *p53* mutations have essentially opposite effects on the male germline following dicentric chromosome induction, since in the soma they both permit survival of cells that would otherwise succumb to apoptosis. This seeming conflict might be resolved by consideration of another function of P53 in somatic cells — its role in compensatory cell proliferation. Cell death that causes significant depletion of the cells in an imaginal disc can be compensated by extra rounds of division of the remaining cells, a process which requires P53 [42], [43]. In the testis, dicentric induction results in transient depletion of germline cells. Our results show that recovery from this depletion also requires P53. We hypothesize that the role of P53 in the male germline is most similar to its role during compensatory cell proliferation in the soma. This might also account for the reduced rate of healing seen with *p53* mutants. Compensatory cell proliferation invokes P53-dependent cell cycle delays [42]. If, as discussed above, healing is a time-dependent process, then lack of P53-mediated cell cycle delays might account for the reduced rate of healing seen in *p53* mutants. In wildtype males, cells with an unrepaired DSB may first experience a cell cycle delay that gives opportunity for healing to occur, albeit infrequently, prior to elimination of that cell. If this delay does not occur, the probability of healing would be reduced even further.

P53 and its relatives, including P63 in mammals, are known to play a variety of roles in the male germlines of several species, including negative regulation of the early germ cell population [44], [45], and positive regulation of cell death in response to DNA damage [46]–[49]. Overexpression of *p53* can also cause apoptosis and germline elimination in otherwise wildtype *Drosophila* males [50]. Our results reveal a new function for P53 in the male germline of *Drosophila*: it is required to re-populate the germline following elimination of cells with a broken chromosome.

In a different experimental paradigm, mutations in DNA repair genes and checkpoint genes were found to increase the frequency of *de novo* telomere formation at an I-*Sce*I-generated cut, although *lok* was not tested [34]. A moderate increase in healing in *p53* mutant males was also observed in those experiments, while we saw a decrease. This may be due to fundamental differences between the two assays. In the experiments of Beaucher *et al.*, the I-*Sce*I cut site was located on an autosome: after cleavage the cells have at least two broken chromosome ends, they carry a homologous chromosome, and in G2, a sister chromatid. In our experiments dicentric bridge breakage during mitotic anaphase produces cells with only a single broken end, and no sequence-matching homolog (because it is the *Y* chromosome) or sister chromatid (at least initially). It is reasonable to suspect that the configuration of homologous sequences and number of broken ends may effect different outcomes in the two sets of experiments. It may be particularly significant that end-joining is a repair option following I-SceI cleavage, but not following dicentric bridge breakage. When the ability to rejoin the ends generated by I-*Sce*I cleavage is reduced or eliminated by mutations in DNA repair genes, healing is the only option that remains for cells to survive, and therefore increases in frequency when compared to the controls. On the other hand, in our experiments, healing is the only available option that allows transmission and recovery of the broken chromosomes.

We envision the following scenario to explain our experimental observations. In wildtype males, a dicentric bridge generated in stem cells or early spermatogonial mitoses typically breaks, most often resulting in Chk2-mediated elimination of cells that inherit the broken fragments. Because dicentric formation in our experiments is very efficient (>90%), this often produces sterility owing to a complete loss of germline stem cells. However, if any germ cells survive, the germline may be re-populated through a mechanism that requires P53. The survivors may be infrequent cells that did not experience dicentric formation or cells in which a broken chromosome has been healed by *de novo* addition of a telomere cap. Surviving cells continue to divide and produce many functional sperm. We suppose that healing is relatively rare in wildtype males, and such males mostly owe their fertility to the few percent of cells that escape dicentric formation. Although the transmission of healed chromosomes from wildtype males is only ∼11%, the males that do transmit healed chromosomes do so at an average rate of 40%, indicating that the germlines of such males typically derive from only ∼2–3 founder cells, compared to 15–20 normally [44], consistent with our contention that the germlines derive from infrequent survivors.

In *lok* males, absence of the Chk2 checkpoint allows cells with a broken chromosome to continue division unhindered. During pre-meiotic proliferation a broken end may be healed in some cells, but not in others, generating cysts that carry healed or un-healed chromosomes, or a mixture of the two. Chromosomes that have not healed by the time of meiosis are likely to experience end-to-end fusion of the uncapped ends of sister chromatids, resulting in MII dicentric bridges that trigger post-meiotic elimination of *Y*-bearing spermatids. This elimination produces strong meiotic drive.

We found that *lok* is haplo-insufficient in the germline, as it is in the soma [16], so that *lok/+* males transmit *FrY* chromosomes at an intermediate rate. However, these males show no evidence of the meiotic drive that *lok* homozygotes show. Perhaps *lok/+* heterozygotes have a reduced probability of detecting and eliminating cells carrying an unrepaired break during pre-meiotic mitoses, thereby allow an increased rate of healing, but still manage to eliminate most such cells prior to meiosis. In the soma *lok/+* heterozygotes exhibit a similar phenotype following dicentric induction: cells with broken chromosomes can persist and form part of the adult wing if they are generated 1–2 days before differentiation, but if they are generated earlier in development they are efficiently eliminated [16].

The best known case of meiotic drive in *Drosophila*, that wrought by the Segregation Distorter (SD) system, also results from the post-meiotic elimination of spermatids [51]–[54]. The molecular identities of both the driving *Sd* element [55] and the *Rsp* susceptibility element [56] are known, but the ultimate cause of spermatid dysfunction is still a mystery. The identification of *Sd* as a truncation allele of a gene encoding RanGap protein placed the focus on nuclear transport [57], [58]. However, no clear mechanisms have emerged from this discovery [59]. Our finding that MII dicentric chromosome bridges are associated with, and almost certainly causative of the meiotic drive in *lok* males is reminiscent of the mechanism proposed for Segregation Distortion in the initial paper by Sandler *et al.* [60]. They suggested that distortion came about when a distorting *SD* chromosome produced a break in a sensitive *SD^+^* homolog in meiosis. The broken ends of sister chromatids would subsequently fuse and produce an anaphase bridge at MII. They proposed that, “Either the bridge itself or a breakage product of it can be imagined to cause the death or nonfunction of the resulting cells; that is, the cells are rendered incapable of proceeding through spermiogenesis.” Although additional genetic evidence in support of such a model was later presented [61], the failure to find cytological confirmation of this mechanism led to it being discounted [62]. In light of our findings here, the proposal by Sandler *et al*. (*ibid.*) seems strikingly prescient. Though our findings do not address whether chromosome breakage is involved in the mechanism of Segregation Distorter, they at least make it clear that such a mechanism can produce meiotic drive.

## Materials and Methods

All flies were raised on standard cornmeal medium at 25°C. The *DcY(H1)* chromosome has been described [12], [16]. The heat-inducible FLP transgenes used in these experiments were: *P{70FLP, ry^+^}3F* [63] and *P{hsFLP, ry^+^}2B* [64]. Heat shocks were applied early in development, since only early stages of spermatogenesis are susceptible to heat shock induction of transcription [65], [66]. Two heat shock protocols were used that differed by when the heat shock was applied. Parents were placed in a vial, and allowed to lay eggs for either 24 hours or 72 hours. The parents were removed and the vials were then heat shocked in a circulating water bath at 38° for one hour and returned to 25°.

### Fragment transmission analysis

Single males were generally mated with two females. Progeny were scored through the 18th day after starting the cross. In crosses of *y w/DcY(H1)* males X *y w* females, occasional yellow sons or yellow^+^ daughters, likely arising by nondisjunction and representing less than 1% of all offspring, were excluded from totals. The Mann-Whitney test was used to compare the fragment ratios or sex ratios of individual males from each genotype.

### Egg to adult survival

*w^1118^/DcY(H1); lok^P6^* males were crossed to *y w 70FLP3F; lok^P6^/(lok^P6^* or *Cy*) females, and the progeny were heat-shocked (or not) at 38° for one hour at 0–24 hours of development. The *y w 70FLP3F/DcY(H1); lok^P6^* males that eclosed were mated to *y w* female virgins. Eggs were collected for 24 hours on standard food and counted. All adults eclosing through the eighteenth day after starting the egg collection were scored.

### Scoring primary spermatocyte cysts after dicentric induction

For analysis of primary spermatocytes in wildtype males after dicentric induction *y w*/*DcY(H1)* or *y w*/*DcY(H1); hsFLP2B/S^2^ CyO* males were crossed to *y w*; *hsFLP2B/S^2^ CyO* females. For examination of *p53, y w/Dcy(H1); hsFLP2B/CyO, GFP; p53^5A-1-4^* males were crossed to *y w; p53^5A-1-4^* females. To examine *lok* males *w^1118^/DcY(H1); lok^P6^* males were crossed to *y w 70FLP3F; lok^P6^/CyO, GFP* females. Eggs were collected for 2–5 days and the vials were heat-shocked for 1 hr at 38° when pupae were present. Sons carrying *hsFLP* or *70FLP*, and eclosing at different times after heat shock, were dissected in 1× PBS. To aid in visualizing primary spermatocyte cysts, testes were treated for 5′ in hypertonic solution (5× PBS), then mounted in 1× PBS and examined with phase contrast optics.

### Examination of meiotic figures and spermatid differentiation

Crosses were started to generate males of the appropriate genotypes, and their progeny were heat-shocked using the 24 hour collection protocol. Testes were dissected from adult males within 24 hours of eclosion, fixed in 1× PBS + 4% paraformaldehyde and then stained with DAPI and phalloidin coupled to FITC or rhodamine. Testes were mounted in 50% glycerol + antifade and examined with an Olympus DSU microscope using Slidebook 5.0 software. When examining sperm head location, we counted a sperm head as displaced caudally from the bouquet if it was separated by the length of at least one sperm head. (Most displaced sperm heads showed much greater separation than this.) To facilitate scoring sperm heads in individual cysts the testis sheath was torn with forceps and, after placing a coverslip on the sample, it was tapped gently to release and spread the contents. Meiotic figures were scored in intact testes. A 2×2 contingency test was used to compare the number of testes found in wildtype vs. *lok* males. The Mann-Whitney test was used to compare the number of MII bridges found in wildtype vs. *lok* testes.

### Transmission of long vs. short fragments

We recovered multiple *FrY* chromosomes from individual males and crossed them to *eyFLP* females to determine whether they were long or short fragment chromosomes. Long fragments carry inverted *FRT*s and undergo FLP-mediated recombination to generate dicentric chromosomes and produce small, rough eyes in the sons of this cross. Short fragment chromosomes, which do not carry *FRT*s, produce normal eyes. To determine whether the distribution of long and short fragments from individual males was non-random we performed 1000 randomization trials using Microsoft Excel, and scored the number of trials that produced an equal or greater number of males with only a single type of *FrY* to determine the probability of such a distribution occurring by chance.

### Effect of maternal genotype on recovery of *FrY* chromosomes

Heat-shocked *y w 70FLP3f•YL, B^S^/DcY(H1)* males were crossed to either *y^1^ w^1118^* females, or *y^1^ w^1118^; lok^p6^* females, or *y^1^ w^1118^; p53^5A-1-4^* females and progeny scored to measure transmission of broken-and-healed chromosomes.
